# Body mass index and cancer risk among Chinese patients with type 2 diabetes mellitus

**DOI:** 10.1186/s12885-018-4675-0

**Published:** 2018-08-06

**Authors:** Hui-lin Xu, Min-lu Zhang, Yu-jie Yan, Fang Fang, Qi Guo, Dong-li Xu, Zuo-feng Zhang, Fen Zhang, Nai-qing Zhao, Wang-hong Xu, Guo-you Qin

**Affiliations:** 1 School of Public Health, Fudan University, 138 Yi Xue Yuan Road, Shanghai, 200032 People’s Republic of China; 2 Shanghai Minhang Center for Disease Control and Prevention, 965 Zhong Yi Road, Shanghai, 201101 People’s Republic of China; 3 Department of Epidemiology, UCLA Fielding School of Public Health, Los Angeles, CA USA; 4 Shanghai Municipal Center for Disease Control and Prevention, 1380 West Zhong Shan Road, Shanghai, 200336 People’s Republic of China; 5 Key Laboratory of Public Health Safety, Fudan University, Shanghai, People’s Republic of China; 6 Collaborative Innovation Center of Social Risks Governance in Health, Fudan University, Shanghai, People’s Republic of China

**Keywords:** Body mass index, Retrospective cohort study, Type 2 diabetes mellitus, Cancer incidence

## Abstract

**Background:**

Obesity and diabetes are two risk factors for cancer. To evaluate the association of body mass index (BMI) with cancer risk in diabetic patients may improve current understanding of potential mechanisms.

**Methods:**

A retrospective cohort study was conducted in 51,004 newly diagnosed T2DM patients derived from an electronic health record (EHR) database of Minhang district in Shanghai, China. Incident cancer cases and all-cause deaths occurred before September 30, 2015 were identified by linking with the Shanghai Cancer Registry and the Shanghai Vital Statistics. To examine the potential non-linear and linear relationships of BMI and cancer risk, Cox proportional hazard models with and without restricted cubic spline functions were used, respectively.

**Results:**

A non-linear association was observed between BMI and overall cancer incidence in men younger than 60 years old (*p* for non-linearity = 0.009). Compared with those having BMI of 25.0 kg/m^2^, the cancer risk increased in those with either lower or higher BMI. In women older than 60 years old, linear dose-response relationships were observed between BMI and the risk of both overall cancer and breast cancer. As each unit increase in BMI, the overall cancer risks elevated by 3% (95%CI: 1–5%) and the breast cancer risks increased by 7% (95%CI: 1–13%). No significant association was observed between BMI and other common cancer sites.

**Conclusions:**

Our results show that the effect of BMI on cancer risk in Chinese patients with T2DM may vary by gender, age and cancer subtypes, suggesting different underlying biological mechanisms.

**Electronic supplementary material:**

The online version of this article (10.1186/s12885-018-4675-0) contains supplementary material, which is available to authorized users.

## Background

The associations of obesity [1–4] and diabetes [5, 6] with cancer risks have recently been drawing much attention, mainly due to alarmingly increasing prevalence of the two chronic conditions [7, 8]. A recent research estimated that 5.6% of all incident cancers in 2012, corresponding to 792,600 new cases, were attributable to the combined effects of diabetes and high body mass index (BMI) [9]. As two independent risk factors for overall and certain types of cancer in general population, diabetes and obesity have common biological mechanisms, such as insulin resistance and hyperinsulinemia [10–13]. However, the nature of type 2 diabetes (T2DM) may differ by BMI, a surrogate marker of leanness/obesity. It is reported that patients underweight or with normal weight may suffer from less beta-cell dysfunction and insulin resistance than those obese [14], while diabetic patients with higher BMI were more likely to experience insulin resistance due to adiposity [15]. T2DM and obesity were also observed to jointly promote the development of certain subtypes of cancer, but the results varied by cancer sites and across populations [16–18].

To evaluate the association of BMI with cancer risk among T2DM patients is another approach to better understanding the mechanisms that link obesity and diabetes with cancers. However, limited evidence is available on the association between BMI and cancer risks in patients with T2DM [19, 20]. In this study, we conducted a large-scale retrospective cohort study based on the diabetes management database in Minhang district of Shanghai, China, to examine the association between BMI and risks of overall and site-specific cancers in Chinese diabetic patients.

## Methods

### Study population

This retrospective cohort study was a population-based study based on a standardized management system of diabetes in Shanghai, China. According to the Chinese National Diabetes Prevention Guide, the standardized management of diabetic patients has been carried out as a basic community health service since 2004 in Minhang district, an administrative area with 1,000,000 residents of Shanghai, China [6, 21]. A total of 52,764 patients were diagnosed with T2DM during the period from 2004 to 2014 based on the 1999 criteria of the World Health Organization (WHO) [22]. All patients were enrolled in this study and followed up until date of cancer diagnosis, death, or September 30, 2015.

### Data collection

Baseline information on demographic factors, diagnosis date of diabetes, self-reported standing height and body weight, and regular exercise was derived from the electronic health record (eHR) database. BMI was calculated as body weight in kilogram divided by squared body height in meters. A total of 1440 patients with any type of cancer at the time of diagnosis of T2DM were excluded, leaving 51,324 (24,124 men, 27,200 women) patients in the study. Patients with incomplete data of BMI (*n* = 229) and those diagnosed with cancer within three months of T2DM diagnosis (*n* = 91) were further excluded. Finally, 51,004 patients (23,981 men and 27,023 women) were included in the analysis.

Outcome of interest in this study was the incidence of any primary cancers. The incident cancers and all-cause deaths in the patients until September 30, 2015 were identified by linking with the Shanghai Cancer Registry and the Shanghai Vital Statistics using a unique identification card number [23, 24]. Cancer cases were ascertained according to the International Classification of Diseases (ICD-10) codes by the type of cancers such as Stomach (C16), Colorectum (C18-C20), Pancreas (C25), Trachea, bronchus and lung (C33-C34), Breast (C50), Prostate (C61), Bladder (C67) and Thyroid (C73).

### Statistical analysis

Person-year (PY) of follow-up was calculated from the date of T2DM diagnosis to the date of diagnosis of primary cancer, date of death, or the end of follow-up (September 30, 2015), whichever occurred first. Incidence rates were calculated as the number of cancer cases divided by the person-years of observation. Comparisons of demographic characteristics and clinical and lifestyle factors across baseline BMI categorized according to WHO classification [25] were assessed using Kruskal-Wallis tests (for continuous variables) or χ^2^ tests (for categorical variables). Log-rank test was used to examine the difference of cancer incidence across groups with different BMI levels.

Cox proportional hazard model was used to estimate the associations between BMI and the risks of both overall and cancer subtypes, adjusting for age at diagnosis of diabetes, comorbidity of hypertension (Yes/No), and family history of diabetes (Yes/No). Patients with unspecified family history of diabetes were treated as a separate group in multivariate analysis. Log-log survival plot was applied to evaluate the proportional hazard assumption for BMI. The potential curvilinear relationship of BMI with cancer risk was examined by utilizing restricted cubic splines (RCS) using the 5th, 50th and 95th percentiles as fixed knots [26, 27]. Two statistical tests were conducted: one was to test the null hypothesis that the regression coefficients of both linear and non-linear terms of the factor were equal to zero, with the result presented as “*p* for overall association”; another statistical test was for the regression coefficient of nonlinear term (i.e. spline variable), with “*p* for non-linearity” < 0.05 indicating a non-linear association. The nature of the relationships was shown visually by figures. Multivariable adjusted hazard ratios (HRs) and 95% confidence intervals (95% CIs) were estimated by Cox regression model with RCS functions using BMI values of 25 kg/m^2^ as reference for any other values of BMI. All analyses were performed using SAS (version 9.4; SAS Institute, Cary, NC). RCS was completed by SAS macro %RCS [28]. All tests were two-sided and *p* < 0.05 was considered as significant.

In sensitivity analysis, a novel E-value approach proposed by VanderWeele and Ding was applied to estimate to what extend unmeasured confounders could explain away the observed association [29]. Moreover, the association between BMI and overall cancer risk was compared with or without the variable of smoking status in male with age younger than 60 years old.

## Results

### Baseline characteristics of T2DM patients

Among newly-diagnosed T2DM patients (*n* = 51,004) with average age of 61.3 years old, 23,981 (47.0%) were men and 27,023 (53.0%) were women. After a total of 324,116 person-year of follow-up (150,646 in men and 173,190 in women), 2764 cancer cases were identified through a record-linkage with the Shanghai Cancer Registry System.

Table 1 shows the demographic characteristics and clinical and lifestyle factors of T2DM patients by baseline BMI. Significant differences were found for age at diagnosis of T2DM, gender, family history of diabetes, pre-existing hypertension, and regular exercise across BMI categories.

**Table 1:** Demographic characteristics, clinical predictors and lifestyle factors in T2DM patients by baseline BMI

Characteristics	BMI (WHO category, kg/m^2^)				*P* values
	< 18.5 (*N* = 1106)	18.5–24.9 (*N* = 29,764)	25.0–29.9 (*N* = 17,578)	> 30.0 (*N* = 2556)	
Diagnosis age of T2DM (x ± SD, yrs)	65.0 ± 12.6	61. 6 ± 11.1	60.6 ± 10.7	60.0 ± 11.2	< 0.001
< 60 years	395(35.71)	13,890(46.67)	8639(49.15)	1293(50.59)	< 0.001
≥ 60 years	711(64.29)	15,874(53.33)	8939(50.85)	1263(49.41)	
Follow-up time (years)	6.42 ± 3.17	6.42 ± 3.10	6.26 ± 3.07	6.05 ± 3.06	< 0.001
Gender (%)					
Male	646(40.3)	14,078(47.3)	8473(48.2)	984(38.5)	< 0.001
Female	660(59.7)	15,686(52.7)	9105(51.8)	1572(61.5)	
Family history of DM (%)					
Yes	237(21.4)	6727(22.6)	4254(24.2)	624(24.4)	0.014
No	695(62.8)	18,781(63.1)	11,127(63.3)	1608(62.9)	
Unspecified	175(15.7)	4256(14.2)	2197(12.5)	325(12.7)	
Pre-existing hypertension (%)					
Yes	647(58.5)	19,859(66.7)	13,544(77.0)	2147(84.0)	< 0.001
No	459(41.5)	9905(33.3)	4034(23.0)	409(16.0)	
Regular exercise (%)					
Yes	260(50.6)	17,918(60.2)	11,004(62.6)	1600(62.6)	0.16
No	296(26.8)	8513(28.6)	5062(28.8)	751(29.4)	
Unspecified	250(22.6)	3334(11.3)	1512(8.6)	204(8)	

### Overall and site-specific cancer incidence across BMI categories

As shown in Table 2, the incidence of overall cancer was 853.5/100,000 in all T2DM patients, 919.6/100,000 in men, and 794.6/100,000 in women. The incidence was higher in underweight (1140.3/100,000) or obese patients (879.6/100,000) than in normal (847.2/100,000) or overweight patients (842.4/100,000). This pattern was more evident in men, among whom the incidence in both underweight (1661.8/100,000) and overweight (871.0/100,000) patients were higher comparing to both normal weight (955.8/100,000) and obese (821.2/100,000) patients (*p* < 0.001). The median follow-up time from T2DM diagnosis to cancer diagnosis was 4.28 and 4.27 years in men and women, respectively.

**Table 2:** Cancer incidence by baseline BMI in T2DM patients

Characteristics	BMI (WHO category, kg/m^2^)					*P* values
	< 18.5 (N = 1106)	18.5–24.9 (N = 29,764)	25.0–29.9 (N = 17,578)	> 30.0 (N = 2556)	Overall	
All subjects						
No. of Subjects	1106	29,764	17,578	2556	51,004	
Person-years	7203.5	191,223.9	110,046.6	15,641.9	324,115.9	
No. of cancer Cases	81	1620	927	136	2764	
Incidence (per 100,000 person-years)	1140.3	847.2	842.4	879.6	853.5	0.069
Male						
No. of Subjects	446	14,082	8468	985	23,981	
Person-years	2948.6	89,664.6	52,242.8	5970.3	150,826.4	
No. of cancer Cases	49	857	429	52	1387	
Incidence (per 100,000 person-years)	1661.8	955.8	821.2	871	919.6	< 0.001
Female						
No. of Subjects	660	15,682	9110	1571	27,023	
Person-years	4255	101,559.3	57,803.7	9671.6	173,289.5	
No. of cancer Cases	32	763	498	84	1377	
Incidence (per 100,000 person-years)	752.1	751.3	861.5	868.5	794.6	0.073

Table 3 presents the incidence of several common cancer types in diabetic patients by BMI categories. Colorectal cancer ranked first in the number of cancer cases and incidence, followed by trachea, bronchus and lung cancer, stomach cancer, female breast cancer, and prostate cancer.

**Table 3:** Incidence of common site-specific cancers in Chinese diabetes patients by baseline BMI

Subtypes of cancer	ICD (10th)	No. of cases	Incidence (1/100,000)	By BMI								
				< 18.5		18.5–24.9		25.0–29.9		> = 30		*P* values
				No. of cases	Incidence (1/100,000)	No. of cases	Incidence (1/100,000)	No. of cases	Incidence (1/100,000)	No. of cases	Incidence (1/100,000)	
Stomach	C16	279	86.08	11	152.7	153	80.01	101	91.78	14	89.5	0.157
Colorectal	C18-C20	451	139.15	10	138.8	266	139.1	148	134.49	27	172.61	0.645
Pancreas	C25	137	42.27	3	41.63	87	45.5	41	37.26	6	38.36	0.758
Trachea, bronchus & lung	C33–34	408	125.88	15	208.12	267	139.63	110	99.96	16	102.29	0.005
Breast ^a^	C50	258	79.6	5	69.36	139	72.69	92	83.6	22	140.65	0.129
Prostate ^b^	C61	129	39.8	3	41.61	74	38.7	49	44.53	3	19.18	0.711
Bladder ^b^	C67	57	17.59	3	69.33	28	16.73	22	26.35	4	25.57	0.114
Thyroid ^a^	C73	115	35.48	3	41.59	62	37.13	48	51.8	2	31.97	0.113
Others		930	286.93	28	394.17	544	284.48	316	287.15	42	271.64	0.404

^a^ among women only; ^b^ among men only

### Non-linear associations of BMI with overall and site-specific cancer incidence among T2DM patients

**Fig. 1:**
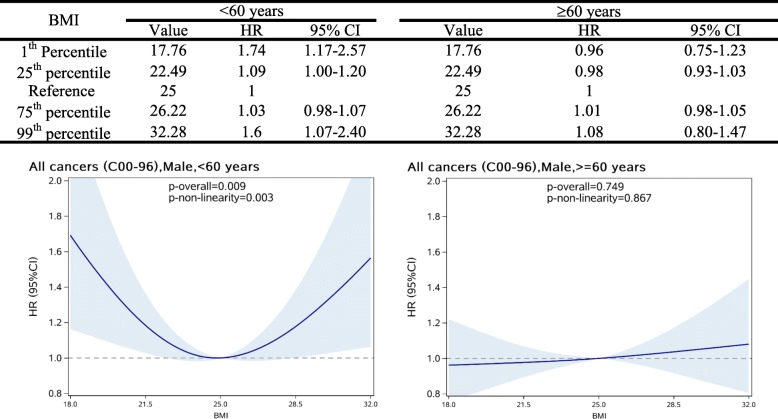
HRs (95%CIs) between BMI (kg/m^2^) and the risk overall cancer in male T2DM patients by age subgroups, allowing for non-linear effects. The reference BMI for these plots (with HR fixed as 1.0) was 25 kg/m^2^

**Fig. 2:**
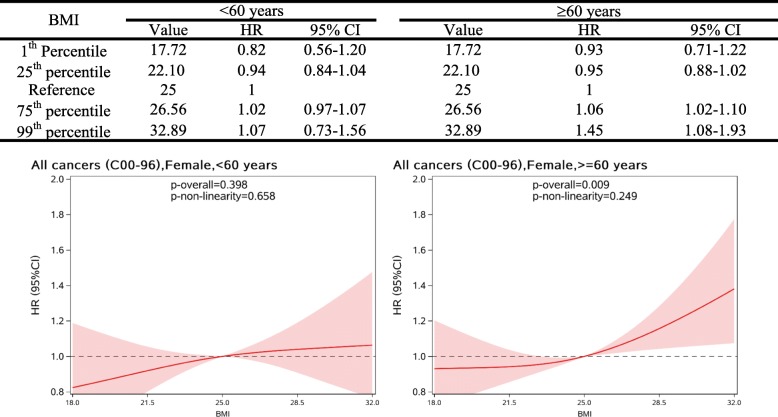
HRs (95%CIs) between BMI (kg/m^2^) and the risk overall cancer in female T2DM patients by by age subgroups, allowing for non-linear effects. The reference BMI for these plots (with HR fixed as 1.0) was 25 kg/m^2^

Figure 1 demonstrates the associations between BMI and overall cancer risk in diabetes patients by sex and age group (< 60 years and ≥60 years). BMI was significantly related to the incidence of overall cancer in men with age younger than 60 years old (*p* for overall association = 0.009). This association was in a non-linear pattern, with an increased risk of overall cancer observed among patients with either lower or higher BMI (*p* for non-linearity = 0.003). Compared with patients with BMI of 25.0 kg/m^2^, those with BMI at the 1st percentile (near 18.0 kg/m^2^) had a 74% increased risk (95% CI: 1.17–2.57) and those at the 99st percentile (near 32.0 kg/m^2^) had a 60% increased risk (95% CI: 1.07–2.40). However, there was no similar association in men older than 60 years old (*p* for overall association = 0.749). In women, BMI was only significantly related to the risk of overall cancer (*p* for overall association =0.009) for those with age older than 60 years old in a linear pattern (*p* for non-linearity = 0.249) as shown in Fig. 2.

**Fig. 3:**
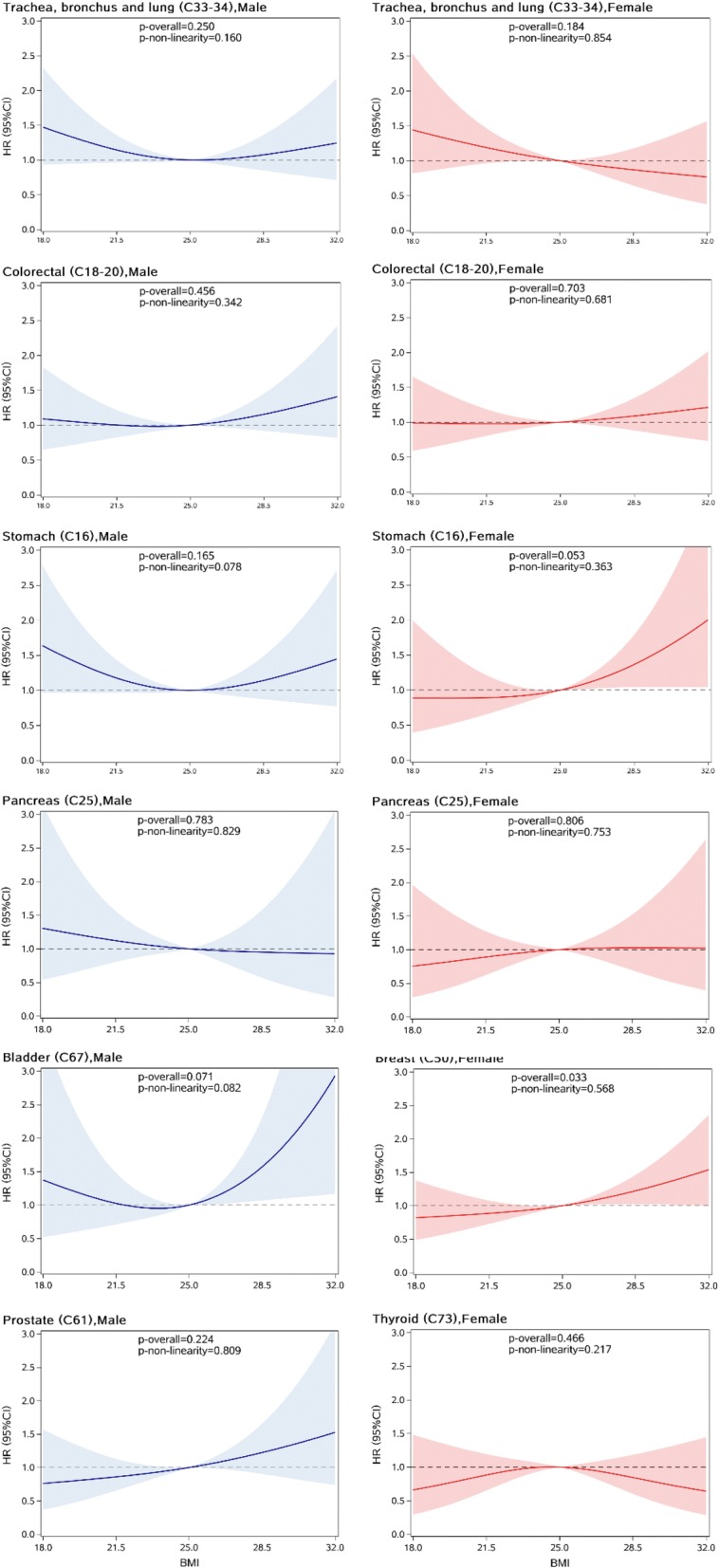
HRs (95%CIs) of BMI (kg/m2) with the risk of common site-specific cancers in male (left) and female (right) T2DM patients, allowing for non-linear effects. The reference BMI for these plots (with HR fixed as 1.0) was 25 kg/m2

As shown in Fig. 3, no significant association was observed between BMI and the risk of any subtype of common cancers like stomach, colorectal, pancreas, lung, bladder, and prostate cancer in men. Also, no significant association was observed between BMI and cancers of stomach, colorectal, lung, pancreas and thyroid in women. However, a significant and linear association was observed between BMI and the risk of breast cancer (*p* for overall association = 0.033, *p* for non-linearity = 0.568). For women older than 60 years old, each unit increase in BMI was linked with a 3% (95%CI: 1–5%) increased risk of overall cancer and a 7% (95%CI: 1–13%) increased risk for breast cancer (Table 4). A similar association pattern was observed for overall and breast cancer when BMI was treated as a categorical variable. Compared with normal weight group (BMI =18.5–24.9 kg/m^2^), significant higher risks of overall and breast cancer were observed in the obese group (BMI ≥30.0 kg/m^2^).

**Table 4:** The association of BMI with the risk of selected subtypes of cancer in T2DM patients

Gender	subtypes of cancer	Categories of BMI	< 60 years old			> 60 years old		
			No. of cancer cases	HR	95%CI	No. of cancer cases	HR	95%CI
Male	All sites of cancer	< 18.5	11	1.42	0.78–2.61	38	1.21	0.87–1.68
		18.5–24.9	241	1(ref)		616	1(ref)	
		25.0–29.9	128	0.89	0.72–1.11	301	0.98	0.86–1.13
		≥30.0	23	1.4	0.91–2.15	29	0.98	0.68–1.43
Female	All sites of cancer	BMI as a continuous variable		non-linearity		833	1.03	1.01–1.05
		< 18.5	9	0.95	0.49–1.85	23	0.9	0.59–1.36
		18.5–24.9	295	1(ref)		468	1(ref)	
		25.0–29.9	214	1.22	1.02–1.46	284	1.15	0.99–1.33
		≥30.0	26	0.86	0.58–1.29	58	1.44	1.10–1.90
	Breast cancer	BMI as a continuous variable		non-linearity		123	1.07	1.01–1.13
		< 18.5	1	0.40	0.06–2.90	4	1.38	0.50–3.82
		18.5–24.9	78	1(ref)		61	1(ref)	
		25.0–29.9	46	0.96	0.67–1.39	46	1.36	0.93–2.00
		≥30.0	10	1.2	0.62–2.33	12	2.19	1.18–4.07

#### Sensitivity analysis

According to the E-value approach, if the observed HR of 1.74 in male would be completely due to unmeasured confounder, a 2.87-fold association between unmeasured confounder and overall cancer risk would be required (Fig. 1). Similarly, a 1.21-fold association between unmeasured confounder and cancer risk would be needed to explain away the observed linear dose response association of BMI and overall cancer risk in female with age older than 60 years old. The association between BMI and overall cancer risk with or without adjusting for smoking status remained unchanged in male younger than 60 years old, as presented in Additional file 1: Figure S1.

## Discussion

In this population-based retrospective cohort study, we found that the associations between BMI and cancer risks varied by gender, age groups (< 60 years old and ≥60 years old) and cancer subtypes among Chinese diabetic patients. In men with age younger than 60 years old, the cancer risk increased with either lower or higher BMI comparing to those with BMI of 25.0 kg/m^2^. In women with age older than 60 years old, a linear dose-response relationship was observed between BMI and the risk of both overall and breast cancer.

Obesity [1–4] and diabetes mellitus [5, 6] are two independent risk factors for overall and certain types of cancer in general population. In our previous study, comparing to the local general population, diabetic patients had an increased risk of overall cancer in both sexes, as well as increased risks of colon, rectum, prostate, and bladder cancers in men and increased colon, breast, and corpus uteri cancer risks in women [6]. Several studies observed a modifying effect of BMI on the association between diabetes and cancer [15–18]. However, few studies have evaluated the BMI-cancer association among T2DM patients and the conclusion was inconsistent.

A register-based cohort study in Sweden has reported that excess body weight was associated with increased risks of all cancer, gastrointestinal cancer and colorectal cancer in male T2DM patients and with elevated risks of all cancer, gestational cancer and postmenopausal breast cancer in female T2DM patients [20]. However, the study did not include patients with BMI less than18.5 kg/m^2^. A study based on the Japan National Center Diabetes Database did not find a significant association between BMI categories and the risk of overall cancers and obesity-related cancers among male patients, but observed a significantly higher risk of overall cancer among female patients with BMI less than 22 kg/m^2^ [19].

We found that the association between BMI and cancer in people with T2DM depend on sex-specific age subgroups (< 60 years old and ≥60 years old). Due to the application of RCS, nonlinear associations characterized by increased risks of cancer in men with lower and higher BMI was observed when comparing with those having BMI of 25.0 kg/m^2^. The results were similar with the results of the Sweden study but inconsistent with the Japanese study. In women older than 60 years old, higher BMI values were related to a higher overall cancer risk, which was consistent with the study in Sweden, but differed from the higher cancer risk that observed in Japanese T2DM women with lower BMI. This controversial association was likely due to the difference in source population of the studies. Community population based on eHR system was selected in our study, but hospital population was recruited in the above mentioned Japanese study. Moreover, ethnic background [4], different subtype of cancers [16, 17], and relevant small sample size of the Japanese study may also contribute to the discrepancy.

Regarding to cancer subtypes, a significant association was observed only for breast cancer risk in female. Our findings are somewhat supported by the results of the Sweden study. The increased risk of breast in female with age older than 60 years is also consistent with a previous study conducted in the general population, in which obese (BMI > 30 kg/m^2^) Chinese women had 36% increased risk of overall cancer and 143% increased risk of postmenopausal breast cancer compared to those with BMI of 18.5–22.9 kg/m^2^ [30]. The difference of BMI and the risk of overall and breast cancer by the age group in female may suggest the effect modification of menopause.

The World Cancer Research Fund reported that being overweight or obese is related to an increased incidence of stomach, colorectal, pancreatic and kidney cancer in both men and women, as well as an elevated risk of advanced prostate cancer in men [31]. For lung cancer, increasing BMI is a protective factor [32]. Different proportion of obesity-related and non-obesity-related cancers in both sexes may contribute to the different association between BMI and the overall cancer risk. However, we did not observe a significant association of BMI with any of these site-specific cancers. The moderate sample size and small number of site-specific cancer cases may lead to limited power for estimating these associations. Ethnicity may also explain the difference of obesity effect between our study and other studies. A positive association of BMI was observed with rectum cancer in European and Australian populations and with pancreatic cancer in North American, but not in Asia-Pacific populations [4]. Further studies are warranted to confirm the observed null associations between BMI and the risk of cancer subtypes observed in our study .

Smoking status may be a potential confounder or an effect modifier. Because about 70% of subjects were also diagnosed with hypertension, we collected smoking information through their hypertension file. For these subjects, the smoking rate for women was only 0.85%. It was unlikely that smoking status biased our estimated association between BMI and cancer in women. The smoking rate for men was about 40%. However, sensitivity analysis suggested that smoking may not be a confounding factor in this study. For other unmeasured confounders, sensitivity analysis with E-value was applied to assess the robustness of the observed association. Even though we do not have these measurements, it is unlikely that these variables would have an effect on cancer risks strong enough to explain away the observed association.

Obesity and type 2 diabetes are closely associated with metabolic abnormalities and poor glycemic control [33, 34] that may contribute to cancer progression [35]. However, the potential mechanisms of obesity, diabetes, and cancer are not yet clear. Many possible explanations have been proposed for certain cancers: The change of hormonal system in insulin, insulin like growth factors, estrogens and other cytokines could be induced by diabetic condition, which may affect the breast cancer risk; the increased production of leptin and the decreased production of adiponectin caused by both obesity and T2DM may cause similar risks for breast cancer [15]. Obesity is being increasingly recognized as sub-clinical inflammation and accordingly contributes to the increase of adipose tissue infiltration of inflammatory components including interleukin-6 (IL-6), tumor necrosis factor-alpha (TNF-α) and C-reactive protein (CRP), all of which have shown to be associated with the development of breast cancer etiology [36]. Other possible mechanisms being explored include the contribution of lipids to cancer development and metabolism, the role of the insulin receptor signaling in cancer, the composition of advanced glycation end products, the changes in hormonal systems to female malignant tumor and growth-promoting effects of obesity and type 2 diabetes on different site-specific cancers. Therefore, the mechanisms underlying the associations between obesity and cancers in diabetic patients are complex and heterogeneous.

The strengths of this study include the retrospective cohort study design, relatively large sample size (*n* = 51,004), and ability to assess associations wih specific cancer sites. Cox proportional hazard models with restricted cubic spline functions help to test potential non-linear relationship with high statistical power. Stratified analysis by sex and age groups enables us to examine the possible difference in sex-specific associations between BMI and overall cancer risk by removing the confounding effect of age. However, several limitations should be mentioned. First, BMI at diagnosis of T2DM may have been affected by the actual duration of diabetes, which is difficult to acquire. Second, self-reported BMI values were used in this study and the number of subjects with BMI < 18.5 kg/m^2^ or BMI > 30 kg/m^2^ was not enough to have a stable estimation of associations. Third, we did not include information on waist circumference, percentage of body fat, level of blood sugar, or the intake of medications such as metformin, making it impossible to evaluate the potential effect of these variables on the risk of cancer in diabetic patients. Finally, although smoking status was less likely to bias the results, the study could not provide an estimated measure of BMI and cancer risk adjusted for smoking.

## Conclusions

In this population-based retrospective cohort study, we found that the associations of BMI with the overall cancer risk varied by gender, age subgroups (< 60 years old and ≥60 years old) and cancer subtypes among Chinese diabetic patients. This indicates complex and heterogeneous biological mechanisms. Increased risks in younger male patients with either lower or higher BMI and in obese older female patients imply that cancer prevention should be focused on these populations.

## Additional file

Additional file 1: **Figure S1.** HRs (95%CIs) between BMI (kg/m2) and the risk overall cancer in male T2DM patients with age younger than 60 years, and pre-existing hypertension allowing for non-linear effects. The shape of the association between BMI and overall cancer risk was compared with or without the variable of smoking status in male with Pre-existing hypertension, and age younger than 60 years old. The reference BMI for these plots (with HR fixed as 1.0) was 25 kg/m2. Left: No adjustment for smoking; Right: Adjustment for smoking; (the effect of smoking after controlling for other variables, *p* = 0.064). (DOCX 197 kb)

## Abbreviations

95% CIs: 95% confidence intervals
BMI: Body mass index
EHR: Electronic health record
HRs: Hazard ratios
ICD: International classification of diseases
PY: Person-year
RCS: Restricted cubic splines
T2DM: Type 2 diabetes mellitus
