# Supplementary material for: Body mass index and cancer risk among Chinese patients with type 2 diabetes mellitus
Source: BMC Cancer. 2018 Aug 6;18:795. doi: 10.1186/s12885-018-4675-0 (PMC6080536; doi:10.1186/s12885-018-4675-0)

| \|  \| \| --- \| |  |  |  |  | \|  \| \| --- \| |  |  |
| --- | --- | --- | --- | --- | --- | --- | --- | --- | --- |
| Fig.S1 HRs (95%CIs) between BMI (kg/m^2^) and the risk overall cancer in male T2DM patients with age younger than 60 years, and pre-existing hypertension allowing for non-linear effects. The reference BMI for these plots (with HR fixed as 1.0) was 25 kg/m^2^. Left: No adjustment for smoking; Right: Adjustment for smoking; (the effect of smoking after controlling for other variables, p=0.064) | | | | | | | |


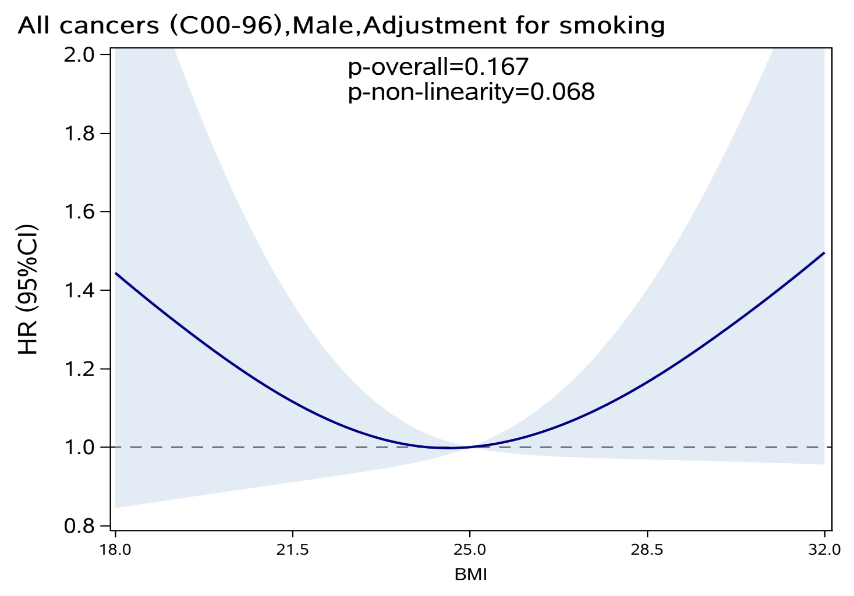

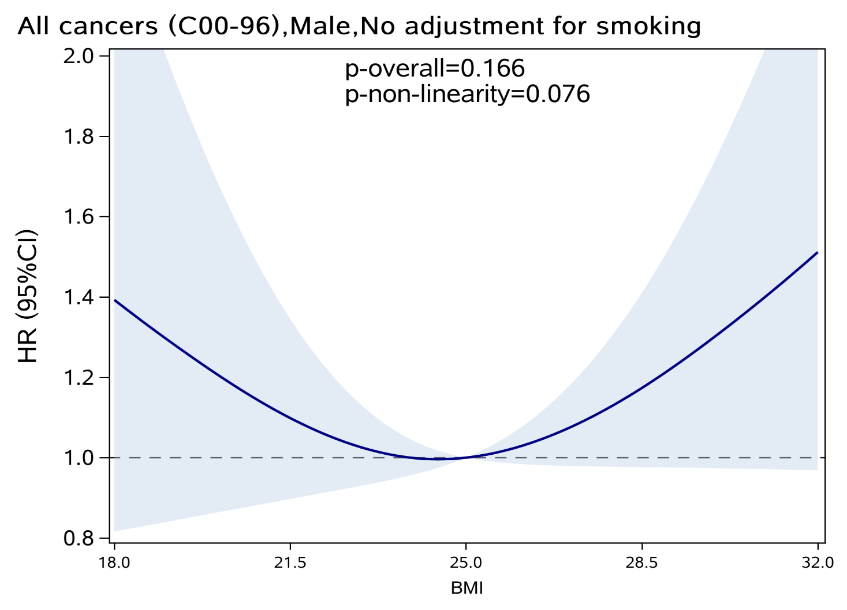

Supplement: Supplementary file 1 — Figure S1. HRs (95%CIs) between BMI (kg/m2) and the risk overall cancer in male T2DM patients with age younger than 60 years, and pre-existing hypertension allowing for non-linear effects. The shape of the association between BMI and overall cancer risk was compared with or without the variable of smoking status in male with Pre-existing hypertension, and age younger than 60 years old. The reference BMI for these plots (with HR fixed as 1.0) was 25 kg/m2. Left: No adjustment for smoking; Right: Adjustment for smoking; (the effect of smoking after controlling for other variables, p = 0.064). (DOCX 197 kb) [file 12885_2018_4675_MOESM1_ESM.docx]
